# Modulation of TLR4/NFκB Pathways in Autoimmune Myocarditis

**DOI:** 10.3390/antiox12081507

**Published:** 2023-07-27

**Authors:** Livia Interdonato, Daniela Impellizzeri, Ramona D’Amico, Marika Cordaro, Rosalba Siracusa, Melissa D’Agostino, Tiziana Genovese, Enrico Gugliandolo, Rosalia Crupi, Roberta Fusco, Salvatore Cuzzocrea, Rosanna Di Paola

**Affiliations:** 1 Department of Chemical, Biological, Pharmaceutical and Environmental Sciences, University of Messina, 98166 Messina, Italy; 2 Department of Biomedical, Dental and Morphological and Functional Imaging, University of Messina, Consolare Valeria, 98100 Messina, Italy; 3 Department of Veterinary Sciences, University of Messina, 98168 Messina, Italy

**Keywords:** experimental autoimmune myocarditis, immunomodulation, oxidative stress

## Abstract

Myocarditis is an inflammatory and oxidative disorder characterized by immune cell recruitment in the damaged tissue and organ dysfunction. In this paper, we evaluated the molecular pathways involved in myocarditis using a natural compound, *Coriolus versicolor*, in an experimental model of autoimmune myocarditis (EAM). Animals were immunized with an emulsion of pig cardiac myosin and complete Freund’s adjuvant supplemented with mycobacterium tuberculosis; thereafter, *Coriolus versicolor* (200 mg/Kg) was orally administered for 21 days. At the end of the experiment, blood pressure and heart rate measurements were recorded and the body and heart weights as well. From the molecular point of view, the *Coriolus versicolor* administration reduced the activation of the TLR4/NF-κB pathway and the levels of pro-inflammatory cytokines (INF-γ, TNF-α, IL-6, IL-17, and IL-2) and restored the levels of anti-inflammatory cytokines (IL-10). These anti-inflammatory effects were accompanied with a reduced lipid peroxidation and nitrite levels and restored the antioxidant enzyme activities (SOD and CAT) and GSH levels. Additionally, it reduced the histological injury and the immune cell recruitment (CD4^+^ and CD68^+^ cells). Moreover, we observed an antiapoptotic activity in both intrinsic (Fas/FasL/caspase-3) and extrinsic (Bax/Bcl-2) pathways. Overall, our data showed that *Coriolus versicolor* administration modulates the TLR4/NF-κB signaling in EAM.

## 1. Introduction

Cardiomyopathies (CMPs) represent a diverse group of heart muscle diseases, defined as myocardial disorders in which the heart muscle is structurally and functionally abnormal in the absence of hypertension, valvular heart disease, congenital heart disease, and coronary artery disease sufficient to explain the observed myocardial abnormality. According to the recent classification of the working group on myocardial and pericardial diseases of the European Society of Cardiology (ESC) [1], they are grouped into specific morphological and functional phenotypes, and each phenotype is then subclassified into familial (genetic) and non-familial (non-genetic) forms.

Myocarditis is a dangerous disease, characterized by inflammation of the heart muscle, which often leads to the development of dilated cardiomyopathy (DCM). It affects approximately 4 to 14 people per 100,000 each year globally and is associated with mortality rates of approximately 1% to 7% [2]. Myocarditis can be classified based on the causative, histological, and clinicopathological criteria [3]. The causative criteria define the infectious agents (virus, protozoa, or bacteria) or non-infectious condition (autoimmune diseases, medications etc.) associated with myocarditis. The clinical manifestations of the disease range from systemic features, such as myalgia, fever, dyspnea, or palpitations, to hemodynamic collapse.

Patients with acute myocarditis should be categorized into complicated or uncomplicated forms of myocarditis. Patients with complicated myocarditis have left ventricular systolic dysfunction, acute heart failure, ventricular arrhythmias, advanced atrioventricular conduction disturbance, or cardiogenic shock. Patients with uncomplicated myocarditis typically present with chest pain and can be treated with nonsteroidal anti-inflammatory drugs (NSAIDs), including aspirin, to relieve chest pain. This wide diversity of clinical symptoms makes the incidence of the disease as well the causes difficult to determine. Several theories have been proposed for drugs, infection, toxic substance exposure, and autoimmune impairments. Recently, the two well described mechanisms have been the progressive autoimmune myocardial injury and viral infection [4]. Frequently, the autoimmune response to myocardial antigens mediates the disease. The pathology is characterized by the recruitment of pro-inflammatory cells in the heart muscle, cardiomyocytes apoptosis, and increased reactive oxygen species (ROS) [5]. These immune-recruited cells promote the secretion of growth factors and cytokines in the interstitial and extracellular spaces [6]. Many papers in particular underline the contribution of immune cells such as monocytes or macrophages and T lymphocytes to cardiovascular disease [7]. For example, Th1 cells are the principal source of the pro-inflammatory mediators IL-2, IFN-γ, and TNF-α, while Th17 is the main source of IL-17. This pro-inflammatory macroenvironment results in the activation of inflammatory signaling, such as TLRs and NF-κB, which exacerbate myocardial injury.

Previous studies indicated that the over-activation of TLR4 was involved in the progression and development of cardiovascular diseases including cardiac hypertrophy, cardiac dysfunction [8], heart failure [9], and atherosclerosis [10]. The over-expression of TLR4 in the innate immune system was associated with increased inflammatory mediators.

Tissue damage is worsened by increased ROS levels and impaired antioxidant defense mechanisms. All of these molecular mechanisms culminate in cardiomyocyte apoptotic death and organ dysfunction.

In the animal model of autoimmune myocarditis, rodents are immunized with cardiac myosin to mimic both the acute phase of myocarditis and chronic phase of DCM in humans [11,12]. Accordingly, 30% of patients with myocarditis and DCM develop high titers of heart-specific autoantibodies [13]. The cardiac myosin heavy chain (MyHC) has been identified as the most prominent autoantigen for circulating heart autoantibodies in myocarditis and cardiomyopathy patients [14]. In fact, the presence of anti-MyHC autoantibodies has been associated with worse left ventricular systolic function and diastolic stiffness in patients with chronic myocarditis [14]. There are strong indications that also antigen-presenting cells play an important role in the pathogenesis of myocarditis in humans by promoting autoimmune mechanisms. For example, a histological analysis demonstrated increased levels of major histocompatibility complex (MHC) classes I and II, known as human leukocyte antigen (HLA) complexes [15], and co-stimulatory molecules B7-1, B7-2, and CD40 [16] in the hearts of myocarditis patient.

Acute myocarditis associated with systemic autoimmune disorders is generally treated with corticosteroids as the first-line therapy, with higher dosages or additional immunosuppressive therapies reserved for patients with a complicated presentation [17]. Unfortunately, when the pharmacological therapy does not work, heart transplantation is the last option. Recently, natural products have assumed important value in the treatment of cardiovascular diseases due to their safety profiles and low prices. Particular attention has been dedicated to mushrooms due to their antioxidant and anti-inflammatory activities [18].

Previous published studies described the potential useful effects of mushrooms including their immunomodulation, antitumor, antibacterial, and antiviral activities [19]. Mushrooms, in fact, are able to stimulate the host’s immune system [19,20]. This ability is due to the high content of β-glucans, which stimulate the cytokine response and activate different types of immune cells. In particular, *Coriolus versicolor* is rich in polysaccharopeptides and β-glucans, hexadecane, vanillic acid, and hexadecanoic acid [21,22]. Several studies, in fact, have reported the antioxidant and immunomodulatory effects of *Coriolus versicolor* and the anti-inflammatory properties as well. Previous studies conducted in our laboratories showed the ability of *Coriolus versicolor* to modulate the TLR4/NF-κB and oxidative pathways in colitis and peripheral multiorgan dysfunction [23,24]. Based on these data, in this paper we evaluated the modulation of this pathway in autoimmune myocarditis.

## 2. Materials and Methods

### 2.1. Animals

Sprague–Dawley rats (200–220 g) were obtained from Envigo (Milan, Italy) and housed in stainless steel cages (3 rats/cage) in a room kept at 22 ± 1 °C and 50 ± 5% humidity with a 12 h dark/light cycle. The animals had ad libitum access to water and standard rodent chow (Teklad standard diet acquire from Envigo). This study was authorized by Messina University’s Animal Welfare Evaluation Board. All studies were conducted in line with new Italian legislation (D.Lgs 2014-26), as well as EU rules (EU Directive 2010-63).

### 2.2. Preparation of Coriolus Versicolor Extract

The Coriolus versicolor biomass was generously donated by Mycology Research Laboratories Ltd. (MRL, Luton, UK). This product is commercially available [25,26].

The characterization was performed using chromatography–orbitrap–mass spectrometry (LC-Orbitrap-MS) and by gas chromatography–tandem mass spectrometry (GC-MS/MS) [27].

### 2.3. Induction of Autoimmune Myocarditis

Equal amounts of purified pig cardiac myosin (Sigma, St. Louis, MO, USA) and complete Freund’s adjuvant (Difco, Franklin Lakes, NJ, USA) supplemented with mycobacterium tuberculosis H37RA (10 mg/mL, Difco) were emulsified. This emulsion was subcutaneously injected in the footpads of rats [28].

### 2.4. Experimental Groups

The rats were randomly divided into the following groups:

- Control: The animals were orally administered with vehicle for 21 days;
- Control + Coriolus versicolor: The animals were orally administered with Coriolus versicolor (200 mg/Kg) for 21 days;
- EAM: The rats were subjected to EAM as previously described and treated
- orally with vehicle every day for 21 days;
- EAM + Coriolus versicolor: The rats were subjected to EAM as previously described and treated orally with Coriolus versicolor (200 mg/Kg) every day for 21 days.

Twenty-one days from the emulsion injection, the animals were sacrificed and organs were harvested fir the histological and molecular analysis.

The route and dose of the Coriolus versicolor were based on previous studies conducted in our [23,24,26] and other laboratories [29]. In previous studies, we already tested the effects of Coriolus versicolor (200 mg/Kg) in different inflammatory models, showing its beneficial effects. In particular, no toxicity was observed, even with prolonged exposure to this compound [23].

### 2.5. Body and Heart Weights

The body weight was monitored during the experiment. At the end the rats were sacrificed and their hearts were weighted to calculate the relative Hw/Bw ratio.

### 2.6. Blood Pressure and Heart Rate Measurements

SBP, DBP, and HR values were measured using an ADistrument BP Blood Pressure Transducer for MLT0699, analyzed using a PowerLab data acquisition system (AD Instruments) and LabChart version 7.2 software [28].

### 2.7. Oxidative Stress Evaluation

The lipid peroxidation was determined as previously described [30]. The level of NO was measured by assaying the total nitrate/nitrite ratio, the stable products of NO oxidation, as described in [31]. The tissue samples were treated with lysate and employed to determine the antioxidant enzyme activity. The CAT determination was carried out using a mixture of CAT buffer and 10 mM of H_2_O_2_, with the detection taking place at 360 nm. The SOD activity was evaluated at 470 nm. The GSH level was determined with 5,5-dithiobis-6,2-nitrobenzoic acid at 420 nm [32].

### 2.8. Cytokines Measurements

The serum cytokines levels were measured using ELISA kits (R&D Systems; Minneapolis, MN, USA; Eagle Biosciences, Inc., Amherst, NH, USA) [33].

### 2.9. RNA Extraction and cDNA Synthesis

An RNeasy kit (Qiagen, Milan, Italy) was employed to extract the RNA for the real-time polymerase chain reaction (RT-PCR) analysis [34]. The quantification was performed RNA with a spectrophotometer (NanoDrop Lite, Thermo Fisher Scientific, Waltham, MA, USA). An iScript RT-PCR kit (Bio-Rad, Hercules, CA, USA) was used to synthesize the first-strand cDNA [35].

### 2.10. Real-Time PCR

In total, 1 μL of total cDNA was used to perform the RT-PCR analysis with the SYBR Green method (Applied Biosystems, Waltham, MA, USA) [36]. GAPDH was employed as an internal control. In addition to biological replicates, three technical replicates were carried out for each target gene. To test for the potential contamination of genomic DNA in the samples, RNA was used as a template for negative controls in all runs.

### 2.11. Histological Analysis

The tissues were harvested and fixed with formalin. They were dehydrated and embedded in paraffin [37]. The tissues slices (7 μm) were stained with hematoxylin and eosin to perform the histological analysis [38,39].

### 2.12. Immunohistochemical Analysis

An immunohistochemical analysis were performed as described previously [40,41]. The heart tissues were fixed in 10% buffered formaldehyde and 7 μm sections were prepared from paraffin-embedded tissues. After deparaffinization, endogenous peroxidase was quenched with 0.3% H_2_O_2_ in 60% methanol for 30 min. The sections were permeabilized with 0.1% Triton X-100 in phosphate-buffered saline (PBS) for 20 min. Non-specific adsorption was minimized by incubating the section in 2% normal goat serum in phosphate-buffered saline for 20 min. Endogenous biotin or avidin binding sites were blocked by sequential incubation for 15 min with avidin and biotin. The sections were probed with the following primary antibodies: anti-CD4 (Santa Cruz Biotechnologies, sc-13573, Dallas, TX, USA) and anti-CD68 (Santa Cruz Biotechnologies, sc-20060). The slides were then washed with PBS and incubated with a secondary antibody. Specific labeling was achieved with an avidin–biotin–peroxidase complex and biotin-conjugated goat anti-rabbit immunoglobulin G (Vector Lab, Milan, Italy) [42]. The stained sections were observed using a Leica DM6 microscope (Leica Microsystems SpA, Milan, Italy).

### 2.13. Western Blot Analysis

A Western blot analysis was performed as already described [43]. Membranes were probed with the following primary antibodies: anti-phospho- IκB (Cell Signaling 2859), anti-MyD88 (Santa Cruz Biotechnologies, sc-74532), and anti-TLR4 (Santa Cruz Biotechnologies, sc-293072) in 1× PBS, 0.1% Tween-20, 5% *w*/*v* non-fat dried milk (PMT) at 4 °C overnight [44,45]. The membranes were incubated with peroxidase-conjugated bovine anti-mouse IgG secondary antibody or peroxidase-conjugated goat anti-rabbit IgG (Jackson ImmunoResearch, West Grove, PA, USA) [46]. The blots were also incubated with the primary antibody against GAPDH (Santa Cruz Biotechnology, Dallas, TX, USA). Signals were detected with an enhanced chemiluminescence detection system reagent according to the manufacturer’s instructions (SuperSignalWest Pico Chemiluminescent Substrate, Pierce, WA, USA) [46].

### 2.14. Tunel Assay

Apoptosis was evaluated using a TUNEL assay (Roche 11684795910, Basel, Switzerland) [47].

### 2.15. Statistical Evaluation

The data are expressed as the means ± SEM from N animals/group. The results were analyzed using a one-way ANOVA, followed by a Bonferroni post hoc test for multiple comparisons. A *p*-value of less than 0.05 was considered significant. Here, * *p* < 0.05 vs. control, # *p* < 0.05 vs. EAM, ** *p* < 0.01 vs. control, ## *p* < 0.01 vs. EAM, *** *p* < 0.001 vs. control, ### *p* < 0.001 vs. EAM.

## 3. Results

### 3.1. Modulation of TLR4/NFκB Pathway

**Figure 1:**
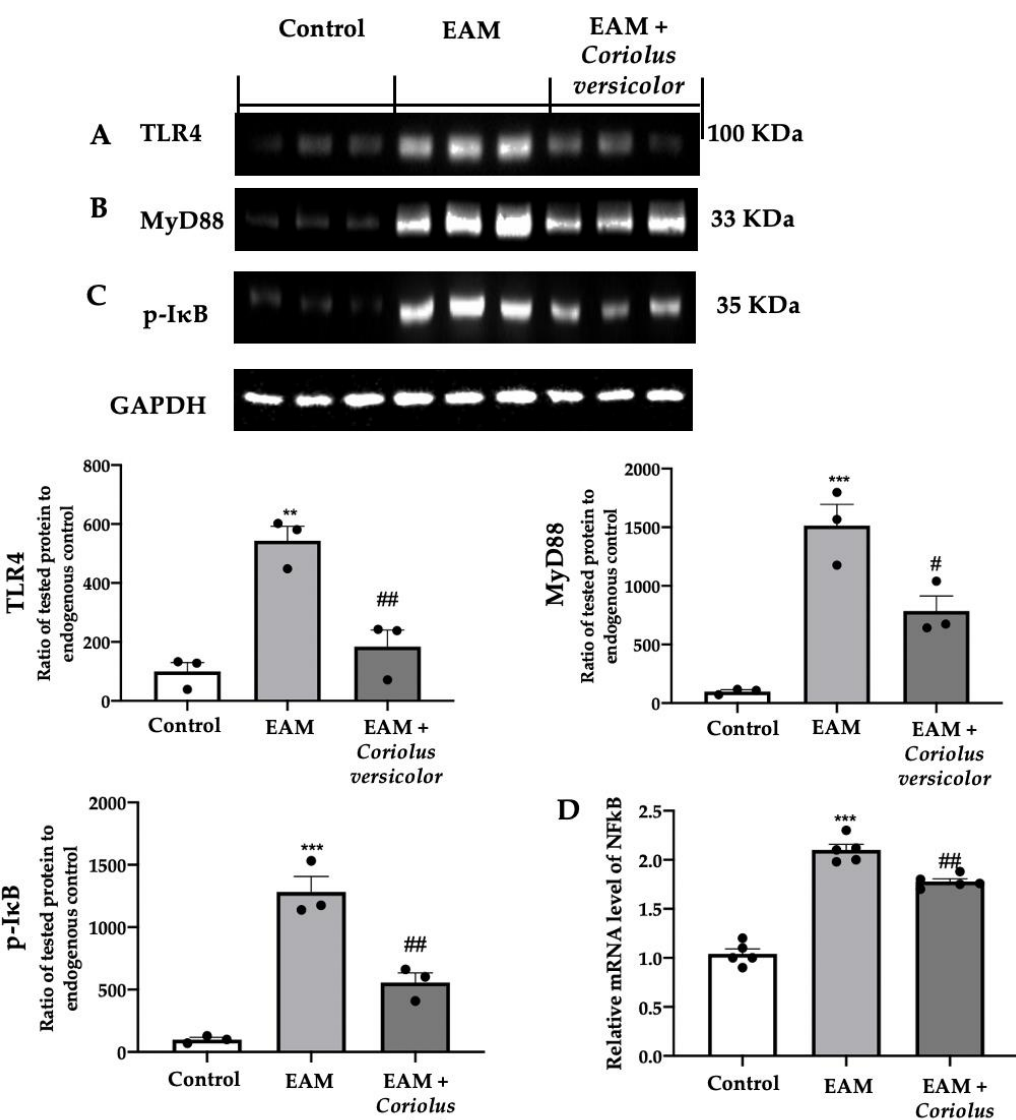
Coriolus versicolor modulates TLR4/NFκB signaling. A Western blot analysis of (**A**) TLR4, (**B**) MyD88, (**C**) p-IkB, and (**D**) RT-PCR with mRNA levels of NFκB. The results were analyzed using a one-way ANOVA followed by a Bonferroni post hoc test for multiple comparisons. A *p*-value of less than 0.05 was considered significant. Note: # *p* < 0.05 vs. EAM, ** *p* < 0.01 vs. control, ## *p* < 0.01 vs. EAM, *** *p* < 0.001 vs. control.

The tissue lysates from the EAM group showed increased TLR4 (Figure 1A) and MyD88 (Figure 1B) expression levels as compared to the control. The Coriolus versicolor reduced both expression levels (Figure 1A,B) in animals with EAM. The Western blot analysis also showed increased IκB phosphorylation in EAM (Figure 1C), while RT-PCR showed increased NFκB levels in the same group (Figure 1D) as compared to the control. The Coriolus versicolor reduced the IκB phosphorylation (Figure 1C) and NFκB mRNA expression levels (Figure 1D).

### 3.2. Modulation of Pro-Inflammatory Cell Recruitment

**Figure 2:**
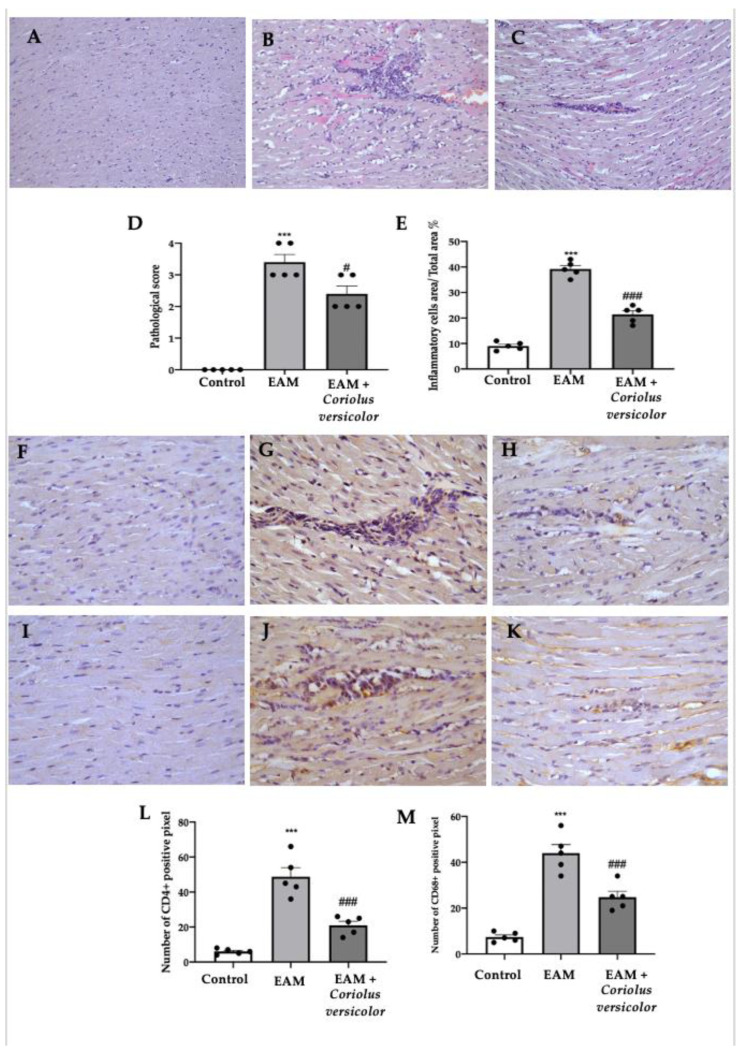
Coriolus versicolor modulates inflammatory cell recruitment. Histological analysis (magnification 20×): (**A**) controls; (**B**) EAM; (**C**) EAM + Coriolus versicolor; (**D**) pathological scores; (**E**) inflammatory cell infiltration scores. Immunohistochemical analysis of CD4 expression (magnification 40×): (**F**) controls; (**G**) EAM; (**H**) EAM + Coriolus versicolor; Immunohistochemical analysis of CD68 expression (magnification 40×): (**I**) controls; (**J**) EAM; (**K**) EAM + Coriolus versicolor; (**L**) graphical quantification of CD4 expression; (**M**) graphical quantification of CD68 expression. The results were analyzed using a one-way ANOVA followed by a Bonferroni post hoc test for multiple comparisons. A *p*-value of less than 0.05 was considered significant. Note: # *p* < 0.05 vs. EAM, *** *p* < 0.001 vs. control, ### *p* < 0.001 vs. EAM.

A histological analysis of the myocardial tissue showed important cellular infiltration and edema in samples collected from the EAM group (Figure 2B,D,E) as compared to the controls (Figure 2A,D,E). Samples from the Coriolus-versicolor-administered animals showed reduced inflammatory cell recruitment and pathological scores (Figure 2C–E). An immunohistochemical analysis was conducted to evaluate the lymphocyte and macrophage infiltration. Increased CD4^+^ (Figure 2G,L) and CD68^+^ (Figure 2J,M) staining levels were found in samples collected from EAM rats as compared to controls (Figure 2F,L,I,M respectively). Coriolus versicolor reduced the CD4^+^ (Figure 2H,L) and CD68^+^ (Figure 2K,M) staining in animals with EAM.

### 3.3. Modulation of Pro-Inflammatory Cytokines and Oxidative Stress

**Figure 3:**
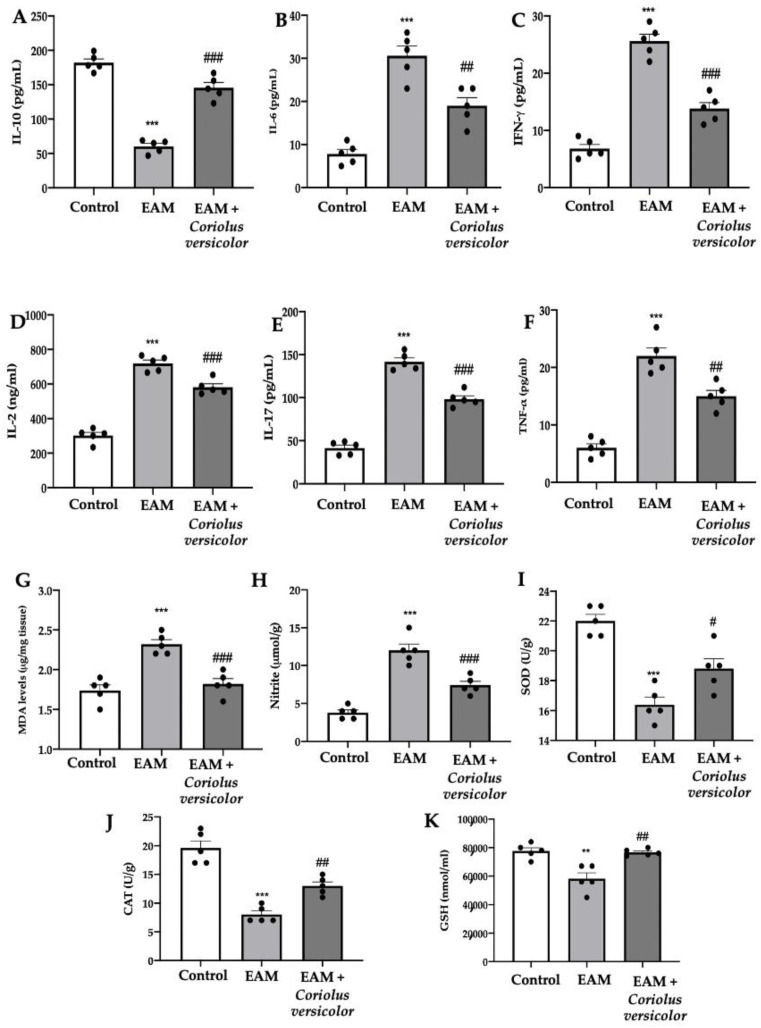
Coriolus versicolor modulates pro-inflammatory cytokines levels and oxidative stress. Serum levels: (**A**) IL-10; (**B**) IL-6; (**C**) IFN-γ; (**D**) IL-2; (**E**) IL-17; (**F**) TNF-α. (**G**) Malondialdehyde (MDA) levels. (**H**) Nitrite levels. (**I**) SOD activity. (**J**) CAT activity. (**K**) GSH levels. The results were analyzed using a one-way ANOVA followed by a Bonferroni post hoc test for multiple comparisons. A *p*-value of less than 0.05 was considered significant. Note: # *p* < 0.05 vs. EAM, ** *p* < 0.01 vs. control, ## *p* < 0.01 vs. EAM, *** *p* < 0.001 vs. control, ### *p* < 0.001 vs. EAM.

The EAM group showed reduced levels of the anti-inflammatory cytokine IL-10 (Figure 3A) and increased levels of the pro-inflammatory cytokines IL-6 (Figure 3B), IFN-γ (Figure 3C), IL-2 (Figure 3D), IL-17 (Figure 3E), and TNF-α (Figure 3F) as compared to the controls. Coriolus versicolor increased the anti-inflammatory and reduced the pro-inflammatory cytokine levels. Coriolus versicolor also caused reduced lipid peroxidation (Figure 3G) and NO release (Figure 3H) as compared to the EAM group. Additionally, Coriolus versicolor restored the SOD (Figure 3I) and CAT (Figure 3J) activities and GSH (Figure 3K) levels, which were reduced by EAM.

### 3.4. Modulation of Apoptosis

**Figure 4:**
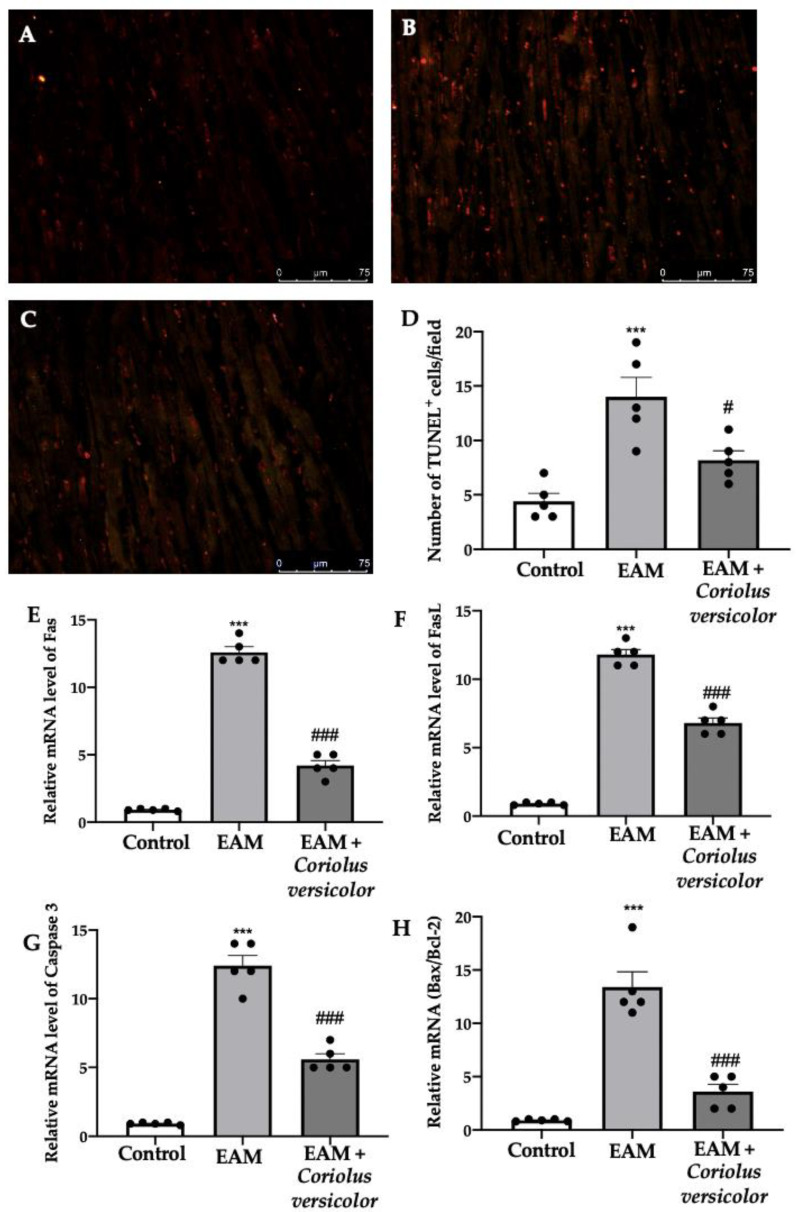
Coriolus versicolor modulates apoptosis. TUNEL assay: (**A**) controls; (**B**) EAM; (**C**) EAM + *Coriolus versicolor*; (**D**) number of TUNEL^+^ cells. RT-PCR for mRNA levels: (**E**) Fas; (**F**) FasL; (**G**) caspase-3; (**H**) Bax/Bcl-2 ratio. The results were analyzed using a one-way ANOVA followed by a Bonferroni post hoc test for multiple comparisons. A *p*-value of less than 0.05 was considered significant. Note: # *p* < 0.05 vs. EAM, *** *p* < 0.001 vs. control, ### *p* < 0.001 vs. EAM.

To identify myocardial cells subjected to DNA fragmentation, a TUNEL assay was performed. Samples harvested from the EAM group showed an increased number of apoptotic cells (Figure 4B,D) as compared to the controls (Figure 4A,D). The Coriolus versicolor administration reduced the number of TUNEL^+^ cells (Figure 4C,D). To further investigate the apoptotic pathways, RT-PCRs were conducted. Increased mRNA levels of Fas (Figure 4E), FasL (Figure 4F), and caspase-3 (Figure 4G) were found in samples from the EAM group as compared to the controls. Moreover, EAM increased the mRNA Bax/Bcl-2 ratio (Figure 4H). The Coriolus versicolor administration reduced the Fas (Figure 4E), FasL (Figure 4F), and caspase-3 (Figure 4G) mRNA levels and the ratio between Bax and Bcl-2 (Figure 4H).

## 4. Discussion

Myocarditis is usually defined as an inflammatory and autoimmune disorder of the heart muscle. Inflammatory cell recruitment has been described as one of the most important characteristics of the pathology, accompanied with reduced cardiac function. This is the reason why this condition can often lead to DCM. The full mechanism of the pathology is not completely known but the increased release of inflammatory mediators and oxidative stress are important actors of the disease. One of the main pathway involved in myocarditis is the TLR4/NF-κB pathway. The enhanced activity of this pathway leads to enhanced cytokine production. Several studies have shown that TLRs and NF-κB signaling are involved in the immunomodulatory effect induced by *Coriolus versicolor* [48,49,50]. In particular, it has been demonstrated that it protects against LPS-induced inflammation caused by these pathways [51,52]. Here, we have shown that *Coriolus versicolor* was able to modulate the TLR/NF-κB pathway also in EAM. TLRs are receptors located on the cell membrane that are activated by specific ligands, and they have a crucial function in initiating the innate immune system and triggering pro-inflammatory pathways.

The binding of TLRs to the adapter molecule MyD88 is widely recognized as the mechanism responsible for attracting monocytes, which then triggers the activation of NF-κB and subsequent signaling cascades. This in turn leads to the generation of various pro-inflammatory mediators such as TNF-α and IL-6, as well as the stimulation of other immune cells [53]. Ultimately, this process initiates local inflammation and the accumulation of leukocytes.

Modulating the NF-κB pathway, *Coriolus versicolor* might play a role in maintaining the equilibrium between Th1 and Th2 inflammatory cytokines, resulting in the alleviation of the inflammatory response in rats suffering from autoimmune myocarditis.

In line with these data, a lower level of inflammatory cell infiltration was found in the myocardial tissues of animals subjected to EAM and administered with *Coriolus versicolor.* Several papers described that T cells and macrophages are crucial immune cells in myocarditis initiation and progression. They are strictly involved in the rich production of pro-inflammatory mediators that contribute to the development of EAM. The pathogenic phases of EAM, in fact, could be divided into three main steps: in the first one, autoreactive T cells are stimulated by fragments of cardiac myosin, which are then recruited to the target organ; the last step involves the effector–target interaction. The cells inducing inflammation are primarily CD4-positive T cells and macrophages [54,55].

Several observations support a role of CD4^+^ T cells as major drivers of autoimmune myocarditis development [56]. During myocarditis induction, various inflammatory cell subsets infiltrate the heart and produce pro-inflammatory cytokines, which create an amplification loop enhancing disease progression. The crucial role of self-reactive CD4^+^ T cells in myocarditis induction is well described, although the mechanisms still remain poorly understood. It is established that IL-17-producing Th17 cells play a major role in the initiation and development of myocarditis. Although both Th1 and Th17 cooperate in disease progression and transition to inflamed DCM, it was claimed that IFN-γ and IL-17 have antagonistic functions in myocarditis and inflammatory cardiomyopathy.

In line with the literature [57], our results showed increased CD4^+^ T cells and CD68^+^ cells in tissues from EAM animals as compared to controls. *Coriolus versicolor* acting on the TLR/NFκB pathway in turn reduced the infiltration of both immune cells, ameliorating the histopathological lesions. These infiltrating cells produce several pro-inflammatory Th17 cytokines (IL-17, IL-6 and TNF-α) and Th1 cytokines (INF-γ and IL-2), resulting in the exacerbation of myocardial injury [55]. Conversely, some already published papers describe the useful effect of IL-10 in myocarditis [58]. IL-10 is an anti-inflammatory mediator produced by monocytes or macrophages and Th2 cells and works as a Th1 cytokine antagonist [59,60]. Its exogenous administration reduced tissue inflammation and reduced inducible NO synthase and pro-inflammatory cytokine secretion, including of INF-γ, TNF-α, IL-6, and IL-2 [61,62]. The modulation of the TLR/NFκB pathway by *Coriolus versicolor* increased the IL-10 levels in animals subjected to EAM, while it reduced the related pro-inflammatory cytokine secretion (INF-γ, IL-6, and IL-2). The literature also shows significant upregulation of IL-17 in this disease [63,64]. The increase in IL-17 levels promotes the expansion of the CD4^+^ Th17 subset [65], while IL-17 antagonists reduce the disease severity [66]. Additionally, the role of TNF-α in myocarditis has been deeply investigated. Its administration results in EAM exacerbation [67], while its suppression or blockage leads to a reduction in cardiac injury [68,69]. As with other natural compounds [55], the *Coriolus versicolor* administration significantly reduced the IL-17 and TNF-α serum levels, demonstrating its anti-inflammatory activity. Moreover, pro-inflammatory cytokines induce myocardial thickness increases and cardiomyocyte contractile impairment, promoting the progression of heart failure [70]. It should be underlined that this pro-inflammatory environment leads to increased ROS levels, worsening the tissue damage [70]. Upregulated NOS-1 expression is related with increased 3-nitrotyrosine formation and lipid peroxidation, which may elicit the formation of peroxynitrite and the production of superoxide [71]. The NO formed by NOS can act on a number of target enzymes and proteins. Carbon monoxide (CO) is another important anti-inflammatory mediator. The most important physiological signaling pathway stimulated by NO and CO is the activation of soluble guanylyl cyclase and the generation of cyclic GMP [72]. As already shown for other natural compounds [32], the modulation of the TLR/NFκB pathway by *Coriolus versicolor* strongly reduced the ROS levels, showing important antioxidant activities. The activation of NF-κB is involved in endothelial dysfunction, hypertrophy, fibrosis, and apoptosis [73,74]. Cardiomyocyte apoptosis is associated with acute and chronic myocarditis and it has been suggested as a causal mechanism of heart failure [75]. Immunomodulatory impairment, increased inflammation, and oxidative stress culminate in myocardial dysfunction and apoptotic cell death [76,77]. It has been already described that natural compounds would be effective in modulating these factors [78]. The mechanism of apoptosis mainly consists of two core pathways involved in inducing apoptosis, namely the extrinsic pathway and intrinsic pathway [79]. The extrinsic pathway refers to the death receptor (DR)-mediated pathway and the intrinsic pathway is a mitochondrial-mediated pathway. Both of these apoptotic pathways, namely the extrinsic and intrinsic pathways, might lead to the same terminal. Apoptotic signaling through the extrinsic pathway is engaged when extracellular ligands such as TNF, Fas-L, and TRAIL (TNF-related apoptosis-inducing ligand) are attached to the extracellular domain of the DR (transmembrane receptors), i.e., the type 1 TNF receptor, Fas (also called CD95/Apo-1) and TRAIL receptors. The order of events involved in the extrinsic phase of apoptosis is well characterized by the FasL/FasR and TNF-α/TNFR1 models [80,81,82]. This triggering of DRs by specific death ligands (DLs) results in the formation of a death-inducing signaling complex (DISC) [83]. This DISC consists of the DD-containing Fas-associated death domain (DD) as an adaptor molecule, procaspase-8, procaspase-10, and the cellular FLICE inhibitory proteins (c-FLIPs).

The intrinsic pathway refers to mainly mitochondrial-mediated apoptotic pathway. The intrinsic pathway is triggered by various extra- and intra-cellular stresses, which include oxidative stress, irradiation, and treatment with cytotoxic drugs [84]. Regarding the pathways of apoptosis, the intrinsic pathway is mediated by Bax/Bak insertion into the mitochondrial membrane, and subsequently cytochrome-c is released from the mitochondrial intermembrane space into the cytosol [85]. Bcl-2 and Bcl-xL (Bcl-2 family member) are antiapoptotic proteins that prevent the release of cytochrome-c. Cytochrome-c combines with Apaf-1 and procaspase-9 to produce the apoptosome. The apoptosome is a multi-protein complex comprised of a seven-spoke ring-shaped complex, which triggers caspase-9 followed by the activation of the caspase-3 signaling cascade, which leads to the demolition of cells and ends in apoptosis.

In our study, we showed that the modulation of the TLR4/NF-κB signaling results in alterations of both the intrinsic (Fas/FasL/caspase-3) and extrinsic (Bax/Bcl-2) apoptotic pathways involved in EAM.

## 5. Conclusions

Nowadays, the treatments for myocarditis are based on the acuity, severity, clinical presentation, and etiology. Therapies with angiotensin-converting enzyme inhibitors, β-blockers, mineralocorticoid receptor antagonists, angiotensin receptor–neprilysin inhibitors, and sodium–glucose co-transporter 2 inhibitors are recommended in patients. In some cases, corticosteroids could be used as a treatment option. If the myocarditis is mild, the cardiologist can prescribe drug therapy combined with bed rest, without hospitalization. In the most serious cases, in patients who do not respond positively to standard treatment, heart transplantation can be considered as a concrete option.

Our study introduces new information about the modulation of the TLR4/NF-κB signaling by *Coriolus versicolor* in EAM. This study has limitations. Further analyses are required to investigate its role in the treatment of myocarditis. This molecular pathway is only one of the many involved in the pathology; thus, the improvement of myocardial functionality and histological injury would be ascribed to cofounding factors.

## Data Availability

The data used to support the findings of this study are available from the corresponding author upon request.

## References

[1] Elliott P., Andersson B., Arbustini E., Bilinska Z., Cecchi F., Charron P., Dubourg O., Kuhl U., Maisch B., McKenna W.J. (2008). Classification of the cardiomyopathies: A position statement from the European Society Of Cardiology Working Group on Myocardial and Pericardial Diseases. Eur. Heart J..

[2] Ammirati E., Moslehi J.J. (2023). Diagnosis and Treatment of Acute Myocarditis: A Review. JAMA.

[3] Blyszczuk P. (2019). Myocarditis in Humans and in Experimental Animal Models. Front. Cardiovasc. Med..

[4] Kawai C. (1999). From myocarditis to cardiomyopathy: Mechanisms of inflammation and cell death: Learning from the past for the future. Circulation.

[5] Rose N.R. (2009). Myocarditis: Infection versus autoimmunity. J. Clin. Immunol..

[6] Camelliti P., Green C.R., Kohl P. (2006). Structural and functional coupling of cardiac myocytes and fibroblasts. Cardiovasc. Gap Junctions.

[7] Amoah B.P., Yang H., Zhang P., Su Z., Xu H. (2015). Immunopathogenesis of Myocarditis: The Interplay Between Cardiac Fibroblast Cells, Dendritic Cells, Macrophages and CD4+ T Cells. Scand. J. Immunol..

[8] Zhao P., Wang J., He L., Ma H., Zhang X., Zhu X., Dolence E.K., Ren J., Li J. (2009). Deficiency in TLR4 signal transduction ameliorates cardiac injury and cardiomyocyte contractile dysfunction during ischemia. J. Cell Mol. Med..

[9] Thomas J.A., Tsen M.F., White D.J., Horton J.W. (2002). TLR4 inactivation and rBPI(21) block burn-induced myocardial contractile dysfunction. Am. J. Physiol. Heart Circ. Physiol..

[10] Edfeldt K., Swedenborg J., Hansson G.K., Yan Z.Q. (2002). Expression of toll-like receptors in human atherosclerotic lesions: A possible pathway for plaque activation. Circulation.

[11] Kodama M., Matsumoto Y., Fujiwara M., Masani F., Izumi T., Shibata A. (1990). A novel experimental model of giant cell myocarditis induced in rats by immunization with cardiac myosin fraction. Clin. Immunol. Immunopathol..

[12] Kodama M., Hanawa H., Saeki M., Hosono H., Inomata T., Suzuki K., Shibata A. (1994). Rat dilated cardiomyopathy after autoimmune giant cell myocarditis. Circ. Res..

[13] Caforio A.L., Mahon N.J., Tona F., McKenna W.J. (2002). Circulating cardiac autoantibodies in dilated cardiomyopathy and myocarditis: Pathogenetic and clinical significance. Eur. J. Heart Fail..

[14] Lauer B., Schannwell M., Kuhl U., Strauer B.E., Schultheiss H.P. (2000). Antimyosin autoantibodies are associated with deterioration of systolic and diastolic left ventricular function in patients with chronic myocarditis. J. Am. Coll. Cardiol..

[15] Herskowitz A., Ahmed-Ansari A., Neumann D.A., Beschorner W.E., Rose N.R., Soule L.M., Burek C.L., Sell K.W., Baughman K.L. (1990). Induction of major histocompatibility complex antigens within the myocardium of patients with active myocarditis: A nonhistologic marker of myocarditis. J. Am. Coll. Cardiol..

[16] Seko Y., Takahashi N., Ishiyama S., Nishikawa T., Kasajima T., Hiroe M., Suzuki S., Ishiwata S., Kawai S., Azuma M. (1998). Expression of costimulatory molecules B7-1, B7-2, and CD40 in the heart of patients with acute myocarditis and dilated cardiomyopathy. Circulation.

[17] Caforio A.L., Pankuweit S., Arbustini E., Basso C., Gimeno-Blanes J., Felix S.B., Fu M., Helio T., Heymans S., Jahns R. (2013). Current state of knowledge on aetiology, diagnosis, management, and therapy of myocarditis: A position statement of the European Society of Cardiology Working Group on Myocardial and Pericardial Diseases. Eur. Heart J..

[18] Elsayed E.A., El Enshasy H., Wadaan M.A., Aziz R. (2014). Mushrooms: A potential natural source of anti-inflammatory compounds for medical applications. Mediat. Inflamm..

[19] Paterson R.R.M., Lima N. (2014). Biomedical effects of mushrooms with emphasis on pure compounds. Biomed. J..

[20] Wasser S. (2014). Medicinal mushroom science: Current perspectives, advances, evidences, and challenges. Biomed. J..

[21] Komura D.L., Ruthes A.C., Carbonero E.R., Gorin P.A., Iacomini M. (2014). Water-soluble polysaccharides from *Pleurotus ostreatus* var. florida mycelial biomass. Int. J. Biol. Macromol..

[22] D’Amico R., Trovato Salinaro A., Fusco R., Cordaro M., Impellizzeri D., Scuto M., Ontario M.L., Lo Dico G., Cuzzocrea S., Di Paola R. (2021). *Hericium erinaceus* and *Coriolus versicolor* modulate molecular and biochemical changes after traumatic brain injury. Antioxidants.

[23] D’Amico R., Tomasello M., Impellizzeri D., Cordaro M., Siracusa R., Interdonato L., Abdelhameed A.S., Fusco R., Calabrese V., Cuzzocrea S. (2023). Mechanism of Action of Natural Compounds in Peripheral Multiorgan Dysfunction and Hippocampal Neuroinflammation Induced by Sepsis. Antioxidants.

[24] Impellizzeri D., Fusco R., Genovese T., Cordaro M., D’Amico R., Trovato Salinaro A., Ontario M.L., Modafferi S., Cuzzocrea S., Di Paola R. (2022). Coriolus Versicolor Downregulates TLR4/NF-kappaB Signaling Cascade in Dinitrobenzenesulfonic Acid-Treated Mice: A Possible Mechanism for the Anti-Colitis Effect. Antioxidants.

[25] Trovato A., Siracusa R., Di Paola R., Scuto M., Ontario M.L., Bua O., Di Mauro P., Toscano M.A., Petralia C.C.T., Maiolino L. (2016). Redox modulation of cellular stress response and lipoxin A4 expression by *Hericium erinaceus* in rat brain: Relevance to Alzheimer’s disease pathogenesis. Immun. Ageing.

[26] Trovato A., Siracusa R., Di Paola R., Scuto M., Fronte V., Koverech G., Luca M., Serra A., Toscano M.A., Petralia A. (2016). Redox modulation of cellular stress response and lipoxin A4 expression by *Coriolus versicolor* in rat brain: Relevance to Alzheimer’s disease pathogenesis. Neurotoxicology.

[27] Cordaro M., Modafferi S., D’Amico R., Fusco R., Genovese T., Peritore A.F., Gugliandolo E., Crupi R., Interdonato L., Di Paola D. (2022). Natural Compounds Such as Hericium erinaceus and Coriolus versicolor Modulate Neuroinflammation, Oxidative Stress and Lipoxin A4 Expression in Rotenone-Induced Parkinson’s Disease in Mice. Biomedicines.

[28] D’Amico R., Fusco R., Cordaro M., Interdonato L., Crupi R., Gugliandolo E., Di Paola D., Peritore A.F., Siracusa R., Impellizzeri D. (2022). Modulation of NRF-2 Pathway Contributes to the Therapeutic Effects of *Boswellia serrata* Gum Resin Extract in a Model of Experimental Autoimmune Myocarditis. Antioxidants.

[29] Chen J., Jin X., Zhang L., Yang L. (2013). A study on the antioxidant effect of Coriolus versicolor polysaccharide in rat brain tissues. Afr. J. Tradit. Complement. Altern. Med..

[30] Di Paola R., Cordaro M., Crupi R., Siracusa R., Campolo M., Bruschetta G., Fusco R., Pugliatti P., Esposito E., Cuzzocrea S. (2016). Protective effects of ultramicronized palmitoylethanolamide (PEA-um) in myocardial ischaemia and reperfusion injury in vivo. Shock.

[31] Abdel-Wahab B.A., Metwally M.E., El-khawanki M.M., Hashim A.M. (2014). Protective effect of captopril against clozapine-induced myocarditis in rats: Role of oxidative stress, proinflammatory cytokines and DNA damage. Chem.-Biol. Interact..

[32] Draginic N.D., Jakovljevic V.L., Jeremic J.N., Srejovic I.M., Andjic M.M., Rankovic M.R., Sretenovic J.Z., Zivkovic V.I., Ljujic B.T., Mitrovic S.L. (2022). Melissa officinalis L. Supplementation Provides Cardioprotection in a Rat Model of Experimental Autoimmune Myocarditis. Oxid. Med. Cell. Longev..

[33] Di Paola R., Fusco R., Gugliandolo E., D’Amico R., Campolo M., Latteri S., Carughi A., Mandalari G., Cuzzocrea S. (2018). The Antioxidant Activity of Pistachios Reduces Cardiac Tissue Injury of Acute Ischemia/Reperfusion (I/R) in Diabetic Streptozotocin (STZ)-Induced Hyperglycaemic Rats. Front. Pharmacol..

[34] Crupi R., Palma E., Siracusa R., Fusco R., Gugliandolo E., Cordaro M., Impellizzeri D., De Caro C., Calzetta L., Cuzzocrea S. (2020). Protective Effect of Hydroxytyrosol Against Oxidative Stress Induced by the Ochratoxin in Kidney Cells: In vitro and in vivo Study. Front. Vet. Sci..

[35] Di Paola D., Capparucci F., Lanteri G., Crupi R., Marino Y., Franco G.A., Cuzzocrea S., Spano N., Gugliandolo E., Peritore A.F. (2022). Environmental Toxicity Assessment of Sodium Fluoride and Platinum-Derived Drugs Co-Exposure on Aquatic Organisms. Toxics.

[36] Di Paola D., Natale S., Iaria C., Crupi R., Cuzzocrea S., Spano N., Gugliandolo E., Peritore A.F. (2022). Environmental Co-Exposure to Potassium Perchlorate and Cd Caused Toxicity and Thyroid Endocrine Disruption in Zebrafish Embryos and Larvae (*Danio rerio*). Toxics.

[37] D’Amico R., Gugliandolo E., Siracusa R., Cordaro M., Genovese T., Peritore A.F., Crupi R., Interdonato L., Di Paola D., Cuzzocrea S. (2022). Toxic Exposure to Endocrine Disruptors Worsens Parkinson’s Disease Progression through NRF2/HO-1 Alteration. Biomedicines.

[38] Hirakawa H., Zempo H., Ogawa M., Watanabe R., Suzuki J., Akazawa H., Komuro I., Isobe M. (2015). A DPP-4 inhibitor suppresses fibrosis and inflammation on experimental autoimmune myocarditis in mice. PLoS ONE.

[39] Zhang Q., Hu L.Q., Li H.Q., Wu J., Bian N.N., Yan G. (2019). Beneficial effects of andrographolide in a rat model of autoimmune myocarditis and its effects on PI3K/Akt pathway. Korean J. Physiol. Pharmacol..

[40] Peritore A.F., D’Amico R., Siracusa R., Cordaro M., Fusco R., Gugliandolo E., Genovese T., Crupi R., Di Paola R., Cuzzocrea S. (2021). Management of Acute Lung Injury: Palmitoylethanolamide as a New Approach. Int. J. Mol. Sci..

[41] Fusco R., Salinaro A.T., Siracusa R., D’Amico R., Impellizzeri D., Scuto M., Ontario M.L., Crea R., Cordaro M., Cuzzocrea S. (2021). Hidrox((R)) Counteracts Cyclophosphamide-Induced Male Infertility through NRF2 Pathways in a Mouse Model. Antioxidants.

[42] Impellizzeri D., Siracusa R., Cordaro M., Peritore A.F., Gugliandolo E., D’Amico R., Fusco R., Crupi R., Rizzarelli E., Cuzzocrea S. (2020). Protective effect of a new hyaluronic acid-carnosine conjugate on the modulation of the inflammatory response in mice subjected to collagen-induced arthritis. Biomed. Pharmacother..

[43] Impellizzeri D., D’Amico R., Fusco R., Genovese T., Peritore A.F., Gugliandolo E., Crupi R., Interdonato L., Di Paola D., Di Paola R. (2022). Acai Berry Mitigates Vascular Dementia-Induced Neuropathological Alterations Modulating Nrf-2/Beclin1 Pathways. Cells.

[44] Genovese T., Impellizzeri D., D’Amico R., Fusco R., Peritore A.F., Di Paola D., Interdonato L., Gugliandolo E., Crupi R., Di Paola R. (2022). Role of Bevacizumab on Vascular Endothelial Growth Factor in Apolipoprotein E Deficient Mice after Traumatic Brain Injury. Int. J. Mol. Sci..

[45] Cordaro M., Fusco R., D’Amico R., Siracusa R., Peritore A.F., Gugliandolo E., Genovese T., Crupi R., Mandalari G., Cuzzocrea S. (2020). Cashew (*Anacardium occidentale* L.) Nuts Modulate the Nrf2 and NLRP3 Pathways in Pancreas and Lung after Induction of Acute Pancreatitis by Cerulein. Antioxidants.

[46] Fusco R., Cordaro M., Siracusa R., Peritore A.F., Gugliandolo E., Genovese T., D’Amico R., Crupi R., Smeriglio A., Mandalari G. (2020). Consumption of *Anacardium occidentale* L. (Cashew Nuts) Inhibits Oxidative Stress through Modulation of the Nrf2/HO-1 and NF-κB Pathways. Molecules.

[47] D’Amico R., Impellizzeri D., Cordaro M., Siracusa R., Interdonato L., Crupi R., Gugliandolo E., Macri F., Di Paola D., Peritore A.F. (2022). Regulation of Apoptosis and Oxidative Stress by Oral Boswellia Serrata Gum Resin Extract in a Rat Model of Endometriosis. Int. J. Mol. Sci..

[48] Engel A.L., Sun G.C., Gad E., Rastetter L.R., Strobe K., Yang Y., Dang Y., Disis M.L., Lu H. (2013). Protein-bound polysaccharide activates dendritic cells and enhances OVA-specific T cell response as vaccine adjuvant. Immunobiology.

[49] Yang S.F., Zhuang T.F., Si Y.M., Qi K.Y., Zhao J. (2015). Coriolus versicolor mushroom polysaccharides exert immunoregulatory effects on mouse B cells via membrane Ig and TLR-4 to activate the MAPK and NF-kappaB signaling pathways. Mol. Immunol..

[50] Price L.A., Wenner C.A., Sloper D.T., Slaton J.W., Novack J.P. (2010). Role for toll-like receptor 4 in TNF-alpha secretion by murine macrophages in response to polysaccharide Krestin, a Trametes versicolor mushroom extract. Fitoterapia.

[51] Jedrzejewski T., Sobocinska J., Pawlikowska M., Dzialuk A., Wrotek S. (2020). Extract from the Coriolus versicolor Fungus as an Anti-Inflammatory Agent with Cytotoxic Properties against Endothelial Cells and Breast Cancer Cells. Int. J. Mol. Sci..

[52] Wang Z., Dong B., Feng Z., Yu S., Bao Y. (2015). A study on immunomodulatory mechanism of Polysaccharopeptide mediated by TLR4 signaling pathway. BMC Immunol..

[53] Kawai T., Akira S. (2007). Signaling to NF-kappaB by Toll-like receptors. Trends Mol. Med..

[54] Barcena M.L., Jeuthe S., Niehues M.H., Pozdniakova S., Haritonow N., Kuhl A.A., Messroghli D.R., Regitz-Zagrosek V. (2021). Sex-Specific Differences of the Inflammatory State in Experimental Autoimmune Myocarditis. Front. Immunol..

[55] Milenkovic M., Arsenovic-Ranin N., Stojic-Vukanic Z., Bufan B., Vucicevic D., Jancic I. (2010). Quercetin ameliorates experimental autoimmune myocarditis in rats. J. Pharm. Pharm. Sci..

[56] Vdovenko D., Eriksson U. (2018). Regulatory Role of CD4(+) T Cells in Myocarditis. J. Immunol. Res..

[57] Schmerler P., Jeuthe S., O h-Ici D., Wassilew K., Lauer D., Kaschina E., Kintscher U., Muller S., Muench F., Kuehne T. (2014). Mortality and morbidity in different immunization protocols for experimental autoimmune myocarditis in rats. Acta Physiol..

[58] Watanabe K., Nakazawa M., Fuse K., Hanawa H., Kodama M., Aizawa Y., Ohnuki T., Gejyo F., Maruyama H., Miyazaki J. (2001). Protection against autoimmune myocarditis by gene transfer of interleukin-10 by electroporation. Circulation.

[59] Yoshida T., Hanawa H., Toba K., Watanabe H., Watanabe R., Yoshida K., Abe S., Kato K., Kodama M., Aizawa Y. (2005). Expression of immunological molecules by cardiomyocytes and inflammatory and interstitial cells in rat autoimmune myocarditis. Cardiovasc. Res..

[60] Chang H., Hanawa H., Liu H., Yoshida T., Hayashi M., Watanabe R., Abe S., Toba K., Yoshida K., Elnaggar R. (2006). Hydrodynamic-based delivery of an interleukin-22-Ig fusion gene ameliorates experimental autoimmune myocarditis in rats. J. Immunol..

[61] Nishio R., Matsumori A., Shioi T., Ishida H., Sasayama S. (1999). Treatment of experimental viral myocarditis with interleukin-10. Circulation.

[62] Yang S., Li W., Liu W., Gao C., Zhou B., Li S., Li Y., Kong Y. (2006). IL-10 gene modified dendritic cells induced antigen-specific tolerance in experimental autoimmune myocarditis. Clin. Immunol..

[63] Sonderegger I., Rohn T.A., Kurrer M.O., Iezzi G., Zou Y., Kastelein R.A., Bachmann M.F., Kopf M. (2006). Neutralization of IL-17 by active vaccination inhibits IL-23-dependent autoimmune myocarditis. Eur. J. Immunol..

[64] Rangachari M., Mauermann N., Marty R.R., Dirnhofer S., Kurrer M.O., Komnenovic V., Penninger J.M., Eriksson U. (2006). T-bet negatively regulates autoimmune myocarditis by suppressing local production of interleukin 17. J. Exp. Med..

[65] Afanasyeva M., Wang Y., Kaya Z., Stafford E.A., Dohmen K.M., Sadighi Akha A.A., Rose N.R. (2001). Interleukin-12 receptor/STAT4 signaling is required for the development of autoimmune myocarditis in mice by an interferon-gamma-independent pathway. Circulation.

[66] Chang H., Hanawa H., Yoshida T., Hayashi M., Liu H., Ding L., Otaki K., Hao K., Yoshida K., Kato K. (2008). Alteration of IL-17 related protein expressions in experimental autoimmune myocarditis and inhibition of IL-17 by IL-10-Ig fusion gene transfer. Circ. J..

[67] Lane J.R., Neumann D.A., Lafond-Walker A., Herskowitz A., Rose N.R. (1992). Interleukin 1 or tumor necrosis factor can promote Coxsackie B3-induced myocarditis in resistant B10.A mice. J. Exp. Med..

[68] Smith S.C., Allen P.M. (1992). Neutralization of endogenous tumor necrosis factor ameliorates the severity of myosin-induced myocarditis. Circ. Res..

[69] Bachmaier K., Pummerer C., Kozieradzki I., Pfeffer K., Mak T.W., Neu N., Penninger J.M. (1997). Low-molecular-weight tumor necrosis factor receptor p55 controls induction of autoimmune heart disease. Circulation.

[70] Varga Z.V., Giricz Z., Liaudet L., Haskó G., Ferdinandy P., Pacher P. (2015). Interplay of oxidative, nitrosative/nitrative stress, inflammation, cell death and autophagy in diabetic cardiomyopathy. Biochim. Biophys. Acta BBA Mol. Basis Dis..

[71] Stockklauser-Farber K., Ballhausen T., Laufer A., Rosen P. (2000). Influence of diabetes on cardiac nitric oxide synthase expression and activity. Biochim. Biophys. Acta.

[72] Forstermann U., Sessa W.C. (2012). Nitric oxide synthases: Regulation and function. Eur. Heart J..

[73] Fiordelisi A., Iaccarino G., Morisco C., Coscioni E., Sorriento D. (2019). NFkappaB is a Key Player in the Crosstalk between Inflammation and Cardiovascular Diseases. Int. J. Mol. Sci..

[74] Lorenzo O., Picatoste B., Ares-Carrasco S., Ramírez E., Egido J., Tuñón J. (2011). Potential role of nuclear factor B in diabetic cardiomyopathy. Mediat. Inflamm..

[75] Kytö V., Saraste A., Saukko P., éronique Henn V., Pulkki K., Vuorinen T., Voipio-Pulkki L.-M. (2004). Apoptotic cardiomyocyte death in fatal myocarditis. Am. J. Cardiol..

[76] Moe G.W., Marín-García J. (2016). Role of cell death in the progression of heart failure. Heart Fail. Rev..

[77] Shimazaki H., Watanabe K., Veeraveedu P.T., Harima M., Thandavarayan R.A., Arozal W., Tachikawa H., Kodama M., Aizawa Y. (2010). The antioxidant edaravone attenuates ER-stress-mediated cardiac apoptosis and dysfunction in rats with autoimmune myocarditis. Free. Radic. Res..

[78] Abukhalil M.H., Althunibat O.Y., Aladaileh S.H., Al-Amarat W., Obeidat H.M., Al-Khawalde A.A.A., Hussein O.E., Alfwuaires M.A., Algefare A.I., Alanazi K.M. (2021). Galangin attenuates diabetic cardiomyopathy through modulating oxidative stress, inflammation and apoptosis in rats. Biomed. Pharmacother..

[79] Jan R., Chaudhry G.E. (2019). Understanding Apoptosis and Apoptotic Pathways Targeted Cancer Therapeutics. Adv. Pharm. Bull..

[80] Elmore S. (2007). Apoptosis: A review of programmed cell death. Toxicol. Pathol..

[81] Jin Z., El-Deiry W.S. (2005). Overview of cell death signaling pathways. Cancer Biol. Ther..

[82] Guicciardi M.E., Gores G.J. (2009). Life and death by death receptors. FASEB J..

[83] Bredesen D.E., Rao R.V., Mehlen P. (2006). Cell death in the nervous system. Nature.

[84] Ghavami S., Kerkhoff C., Los M., Hashemi M., Sorg C., Karami-Tehrani F. (2004). Mechanism of apoptosis induced by S100A8/A9 in colon cancer cell lines: The role of ROS and the effect of metal ions. J. Leukoc. Biol..

[85] Kim R. (2005). Recent advances in understanding the cell death pathways activated by anticancer therapy. Cancer.

